# Hospital Policy in America: Problems of the Future

**Published:** 1916-10-28

**Authors:** Winford H. Smith

**Affiliations:** Superintendent, the Johns Hopkins Hospital, Baltimore, Md.


### HOSPITAL POLICY IN AMERICA.*

### PROBLEMS OF THE FUTURE.

**By Winford H. Smith, M.D.,**

**Superintendent, The Johns Hopkins Hospital, Baltimore, Md.**

The criticism has frequently been made that this Association is not accomplishing all that it should, that the membership should be more representative, and that something radical needs to be done in order to develop our Association and to give it new life. I dare say the Association could accomplish more, but is not that true of any organisation? And is it not indicative of a proper state of mind that we are not fully satisfied? However that may be, it is my profound conviction that the American Hospital Association, by its annual meetings, by the papers and the discussions both in and out of session, and by the enduring friendships formed at the meetings, has had a pronounced effect on the development of the hospital movement in America. I do not share the view that because we have not a larger membership the Association has failed. Indeed, I can well remember that much help and much stimulation were obtainable when the membership was even smaller than it is now.

### Problems of Membership.

Let us consider for a moment the question of membership. A few years ago, the Association was enlarged to take in trustees, members of medical and surgical staffs, and superintendents of nurses. Many of these have availed themselves of the privilege, but in reality only a small percentage of those who are eligible. To me this is neither surprising nor unexpected. Trustees, for the most part, are busy men of affairs; and, while they would no doubt enjoy and profit by attendance at these meetings, I think we can hardly expect them to attend, except those local to the convention city. Physicians and surgeons are already members of so many clinical societies that it is almost impossible for them to attend the meetings of those societies. Furthermore, it is the clinical side of hospital work in which they are primarily interested. Superintendents of nurses have their own Association (the American Nurses' Association, with a membership of some 30,000), which meets annually and provides section meetings and programmes quite as interesting to them as anything which we could offer. It is not surprising that they have not joined our Association in large numbers. We could hardly expect it. In my opinion, so long as we maintain our present organisation, we must expect our membership to be made up primarily of those who are engaged in administrative work—namely, the executive officers.

Why, then, have we not more of the Hospital superintendents on our list? There are several reasons. Undoubtedly there are many who have not yet seen the advantages of exchanging ideas with others engaged in similar work, and who are content to revolve in their own little circles. Many others, if they attend these meetings, must do so at their own expense and during the time allotted to them for vacation. As to those who are so short-sighted as to have no desire to get any new ideas and the point of view of others, I have nothing to say; but for the many who are deterred from joining this Association and attending its meetings on account of the expense and time involved, I feel great sympathy. We must try to help them. This year the Chairman of the Executive Committee, Dr. Washburn, sent out approximately 5,000 letters addressed to Boards of Trustees, urging that their hospital superintendents be sent to this meeting, and setting forth the advantages of membership in this Association. I trust those letters may be fruitful. However, we have no cause to despair because our membership does not grow more rapidly. If our meetings are really helpful and stimulating to the thousand or more members which we have, then a great amount of good is being accomplished for the hospitals of America.

### An Interesting Suggestion.

On the other hand, it is possible that we should consider a change, and I have two suggestions to offer. Might it not be more beneficial in the end if there were formed an Eastern, a Western, a Central, a Southern, and a Canadian Hospital Association, each meeting every two years in its own territory? Then let the American Hospital Association meet in the alternate years, and make membership in the larger Association dependent upon membership in the smaller bodies. The smaller Associations might interest many whom we do not now reach, and by thus stimulating their interest result in a considerable increase in the membership of the larger organisation. I will not dwell longer on this, for the Committee on the Development of the Association may have something to report along this line.

There is another line of thought which has been much in my mind. This is the era of preventive medicine. Public-health work is ever increasing and its scope broadening, and hospitals are each year assuming a more important part in matters relating to the health of the community. Has the time come for the formation of a large American Public Health Association, which would embrace

* Presidential Address at the American Hospital Conference at Philadelphia, 1916.

many and varied activities now represented by independent organisations? Would it be possible to enlist in such a movement such associations, for example, as the American Hospital Association, the present American Public Health Association, the National Association for the Study and Prevention of Tuberculosis, the Canadian Public Health Association, the American School Hygiene Association, the Association for the Prevention of Infant Mortality, the National Organisation for Public Health Nursing, and the National League for Nursing Education? Each Association could preserve its identity as a section of the larger Association, and programmes could be arranged to represent the work of each section. Would not the opportunity thus afforded of following the work of the different sections have a broadening educational influence on all members? Would it not possibly give impetus to the public-health movement? I offer these simply as suggestions for your consideration.

In preparing the programme this year I have been impressed with the fact that we have a large number of committees which, according to the by-laws, are expected to report annually. It is my opinion that, notwithstanding the fact that these reports are invariably interesting, it would be better to eliminate some of them, and leave the selection of the subjects to be presented to the discretion of the President. With this idea in mind, I recommend to you that the Committee on Hospital Progress, which includes a Committee on Efficiency, Finances and Economics, a Committee on Medical Organisation and Education, a Committee on the Training of Nurses, a Committee on Out-Patient Work, and a Committee on Hospital Accounting, be discontinued, with the possible exception of the Committee on Out-Patient Work, but also including the Committee on Legislation and the Committee on the Development of the Association. These committees could be appointed temporarily at any time that the Association might see fit. As it is now, the number of committee reports interferes with the preparation of a well-balanced programme. An amendment to the by-laws is necessary if this recommendation meets with your approval.

Dr. Howell, in his Presidential Address, recommended the formation of a Council or House of Delegates for the transaction of business. The need of this becomes more apparent each year. Too much time is occupied in the transaction of business which could be employed to better advantage for the majority of the members. This matter deserves your consideration.

I wish now to speak briefly of some of the defects in present hospital policy and organisation, and of some of the problems of the future.

### The Hospital Movement.

The growth of the hospital movement has been phenomenal; and, as was to be expected, such rapid growth has developed many weaknesses. It is generally recognised that the modern hospital is a valuable asset to the community. Modern medicine and surgery demand hospital facilities for the successful treatment of many conditions. In almost every hospital there is developed a higher standard each year, necessitating more and better nursing, more refined methods of diagnosis, more extensive equipment, and a larger medical and surgical service. The function of the hospital is too important to be taken lightly, and it is therefore to be regretted that so many hospitals are established as the result of the ill-advised and uncontrolled enthusiasm of groups of individuals who take a too optimistic view of the financial resources of the proposed institution, or who fail to take that point into consideration at all. The result is too frequently a struggling institution, endowed only with the confidence of the public, which it ill deserves. There should be some machinery for the regulating of such enthusiasm. Hospitals without visible means of support should not be allowed to spring up indiscriminately. That many institutions starting from such beginnings have achieved conspicuous success is true, but the principle is wrong, and the practice unsuccessful as applied to the majority. Too frequently such an institution means another unnecessary burden upon the community, diverting funds from more worthy hospitals which are entirely adequate to the community needs.

### The Labourer's Hire.

Another result of this unregulated, mushroom-like growth of hospitals has been continued low standards of service, due in large measure to the low salaries and wages paid. The salaries paid, from the chief executive down, have been and still are altogether too low in the majority of hospitals, and are not at all comparable to the remuneration for less responsible duties in other lines of work. It is difficult to understand how the trustees of hospitals, usually men of keen business ability, can continue to practise a business policy in the conduct of their hospitals which they would not countenance in private business. It is not surprising, therefore, that the work is often below par, and that the changes in *personnel* are so frequent that there are times when but for a few faithful employees the institutions could not operate. It is also not surprising that the standards of decorum and morals are often so low as to amount to scandal. What else could be expected? For example, it is a well-known fact that until recently a large percentage of the employees in some of the municipal hospitals of New York City came from the workhouse on Blackwell's Island.

While on this subject of morals, I wish to say a word more. Recently in discussing the organisation of a new hospital (which is the hobby of one man) with one of its officers, he remarked that Mr. So-and-so did not want the doctors, nurses, and employees to live in the hospital because of the prevalence of immorality under such conditions. We know that such a statement is not true, but the condition does happen just often enough to lend colour to such a statement. Dr. Washburn, in his Presidential Address, touched upon this point, saying: "Well-authenticated report comes to me that in some hospitals in certain sections of the country the standards of deportment, decorum, and in some instances even decency, are low."

Most of us take hospital work seriously. We believe it to be a serious and responsible undertaking. I am sure you will agree with me that no man or woman of low moral standards has any business in a hospital, particularly in a position of trust and responsibility. It should be our concern as members of this Association to see that for the sake not only of the dignity of this Association, but of hospitals generally, such persons are never admitted to membership. I may seem to lay undue emphasis upon this subject, but it only requires a few isolated instances to cast discredit on the entire hospital system.

Taken by and large, hospital organisation is slowly improving, although the old rotating service still prevails, and appointments continue to be made for other reasons than that of merit. I am aware that the problem, particularly in small communities, is a difficult one. The so-called leading physicians and surgeons seem to be necessary to the existence of the hospital, yet, as a matter of fact, a capable man of less reputation, who will take his work seriously and who will give the time which is necessary, is often far more desirable. Social prestige is often a potent factor, although the controlling of hospital appointments by a social clique is an evil.

The continuous service is recognised as much more beneficial to the patients, and much more stable and economical as a working organisation. The readjustment along lines recognised to be for the best interest of the patients, which is the prime consideration, should take place much more rapidly than at present, and should have the backing of this body.

### Merit *versus* Seniority.

Appointments to staff positions should be guarded more carefully, and merit alone should be the determining factor. A long service as an assistant, or in a subordinate position, should not mean promotion unless that service has been of such merit as to warrant it. In hospital work, the best man available, regardless of previous service in that particular hospital, is the man for the place.

While it is true that it costs more each year to operate hospitals, and we often wonder where the money is to come from, nevertheless it must be recognised, and the public should understand, that modern medicine and surgery demand more thorough methods and most elaborate equipment, and if the work is to be properly done these needs must be supplied. In too many hospitals we find inadequate laboratory facilities and incompetent laboratory workers. The *x*-ray laboratory, the clinical and pathological laboratories, properly equipped and in the hands of competent workers, are essential to even, thorough routine work. More attention needs to be given to these points, and I wish to emphasise the fact that in American hospitals too little attention has been paid to the importance of autopsy findings. Furthermore, I think the tendency of hospital administrators has been to surround the autopsy privilege with so much red tape and legal procedure as to be somewhat responsible for the small percentage of autopsies in our institutions—that, and the failure to provide proper machinery for obtaining such permission. Leaving this to inexperienced internes, who are impatient, and sometimes brusque and even threatening in their attitude, is a wrong policy. We are just beginning to realise that investigation and education is a legitimate function of the hospital, quite apart from the beneficial effect that it has upon the patients under immediate treatment.

Another procedure which has been faulty, and still is in too many hospitals, is the manner in which anæsthesia is handled. In the main, anæsthetics are administered by those who are inexperienced and often incompetent. The giving of an anæsthetic is now recognised as an important and it may be a dangerous procedure. To continue to allow this to be done by those who are inexperienced is reprehensible. We should no more allow incompetent anæsthetists than we should allow incompetent surgeons to operate.

### The Civic Status of Hospitals.

The hospitals have rendered a great service to humanity, but in the light of our present knowledge we must recognise that they have been conducted more as repair shops in comparison to what should be the development of the future. We need to study and to understand the broader relationship of the hospital to the community. That we have begun to appreciate this broader usefulness is evidenced by the inauguration of social service work, indicating that we are no longer content to ignore the relationship of the individual to his family, to previous environment, to the community, and to post-hospital environment and opportunities. The pay clinic for people of moderate means, such as has been started at the Massachusetts General Hospital, is also an indication of the growing appreciation of the larger field and the greater possibilities for public service. In the great public-health movement, the hospital, with its opportunities for intensive work, with its trained clinicians and investigators, and its many points of contact with the community, occupies an important, indeed a strategic position. As administrators, we must be something more than good stewards. We must be able to interpret correctly the opportunities open to us for a larger usefulness, for a greater public service.

As an Association, we should endeavour to establish better standards of hospital organisation. One of the fundamental weaknesses in hospital organisation is the too general lack of appreciation of the proper relationships between the trustees and the superintendent, and between the superintendent and the staff. Hardly a day passes that I do not receive letters asking me for advice on this subject, and usually they are from harassed superintendents. There should be no such difficulty. It is a well-recognised business principle that any large organisation must have a single executive head. In a hospital the superintendent is, or should be, that executive head, and the executive officer of the trustees, and the authorised means of communcation between the trustees and all departments. As such, he should have the confidence and backing of the trustees. They should hold him responsible for the management of the institution, and should clothe him with all the necessary authority. He should be subject to their control but not to needless interference. He should in reality be an adviser to the board, and should participate in its deliberations, for he is the one man in the whole organisation who should be best informed on all details pertaining to the administration of the hospital.

The staff has its function—the care of the patients—but that is not the administration. There should not be antagonism between the staff and the superintendent. The staff members should understand that he is not trying to hamper them individually or collectively, but that because of his impartial position, and because of his broader knowledge of affairs in all departments, and of the resources available, he is trying to co-ordinate individual efforts to form a harmonious and efficient machine. If this were understood and practised more generally by both superintendent and staff the efficiency of our hospitals would be markedly increased. With the many splendid examples of hospital organisation available there is scarcely any excuse for the continued haphazard, friction-producing methods so generally practised.

### The Placing of Hospitals.

I have already referred to the evils of unrestricted hospital growth, but there is another phase which should be mentioned, and that is the need of more attention to the location of hospitals with reference to community needs. In the larger cities we not infrequently see hospitals of the same type grouped together in certain sections, while other sections of the city are entirely without hospital facilities. It would be just as sensible if the same policy were followed in locating the public schools. The difficulty lies in the fact that the majority of hospitals are private corporations, and there is no one agency, as in the school system, to pass upon the question of location. There should be some such agency to correlate hospital development with community needs. This point has been well brought out by Mr. Michael Davis in his article on "Health Centres," in which he advocates a hospital, a dispensary, and an open-air school for each 100,000 of the population.

There should be more co-operation between the city authorities and the hospitals in the matter of remuneration of the hospitals by the city for the work done. The city authorities should recognise the extent to which the corporate institutions are shouldering the burden of caring for the sick poor, and should be prepared to recompense those institutions at a rate somewhat approaching the actual cost of such service, and not by the arbitrary and senseless standards which now prevail, representing, broadly speaking, not more than one-half or one-third of the actual cost.

This Association should stand firmly for higher standards in the administration of municipal hospitals, and as unalterably opposed to political domination of those institutions. In this country, the endowed institutions have been the leaders in hospital achievement, while with few exceptions the municipal hospitals have suffered from the political spoils system, with the result that the municipal hospitals in some of the larger cities, which should have been examples of excellence, have in reality been a disgrace to their respective communities. In this connection I need only mention Chicago, New York, Cincinnati, Baltimore, and Philadelphia. We have had a few conspicuous examples of excellence, but they have been all too few; and there is no reason whatever why our municipal institutions, once freed from the pernicious influence of politics, could not be models of excellence, contributing largely to medical education and to the increase of medical knowledge, while at the same time exercising a much more potent influence in maintaining the health of the community. If there is any institution which should be entirely divorced from the evils of political domination it should be these hospitals, which are responsible for the care of the sick poor.

### The Ideal Size for a Hospital.

We have been building hospitals larger and larger, until it is no uncommon thing to find a hospital, located in the heart of a city, which can accommodate from 1,000 to 2,000 patients. It seems to me that we should be planning differently for the future. A large central city plant is expensive to build and expensive to operate. We know that after the acute stages of illness both medical and surgical patients would do quite as well in less elaborate surroundings; indeed, we know that they could be just as well cared for at less expense, and would be infinitely happier, if they could be moved to the country into a convalescent hospital. It might not be overstating the fact to say that under such conditions the period of convalescence might be shortened and the end result bettered. The city plant should, of course, be developed to a point of general usefulness as a general hospital, but beyond that point I believe we should look upon the country branch as the best means of expansion, rather than continue to expand into larger and larger city plants. Furthermore, the lack of facilities for placing patients who need continued convalescent care is a problem confronting hospitals everywhere.

If the hospital system is to be thoroughly efficient, more attention must be paid to the physically handicapped, whom we are constantly discharging from hospitals to lead useless lives, or to return again and again because there is at present no comprehensive plan for placing these individuals in proper environment and for fitting them to lead fairly normal and useful lives. We have some excellent examples both of the need and of the possibilities of such service in Dr. Hall's pottery at Marblehead, in the workshops of the Massachusetts General Hospital and of the Burke Foundation, and in numerous other places. It is my conviction, however, that this is a larger problem, and is not a question to be solved by individual hospitals for their own patients, because that means needless reduplication. Rather, it is a problem of the community. Just as a hospital is a well-organised, well-equipped unit for doing a specific work, so there is needed in every community of considerable size a well-organised, well-equipped unit or organisation for meeting the problem of readjustment —or re-education of those who are physically handicapped, thereby saving many from the useless, dependent, aimless existence to which they are now condemned on account of the lack of some such system. We should be able to learn much from the manner in which this problem is met in England, France, and Germany after the war. On a smaller scale, however, the same problem has been and will continue to be with us, and it must be solved.

### What Hospitals Stand For.

Hospitals were originally designed for the care of the indigent alone, and were looked upon as the places of last resort. With the improved and refined methods of diagnosis and treatment, the more elaborate technique of both medicine and surgery, the hospital is now recognised as the place of first resort for those who are seriously ill. We must therefore realise that the hospital, if it is to be complete and prepared for its broadest usefulness, must provide accommodation for all classes. It is true that American hospitals have been developing along these lines, but it should be emphasised in its importance, else those institutions which provide only for the poor will be in the position of discriminating against the man who can pay, and we shall find the private hospital and the nursing home flourishing as they do elsewhere. Without unduly criticising these institutions, it must be recognised that they cannot afford the advantages of the large general hospital, with its more complete organisation and equipment. Sir William Osler has said that one of the great weaknesses of English hospitals is the lack of proper provision by general hospitals for the well-to-do as well as for the poor, and in England the nursing home flourishes.

Furthermore, one of the greatest needs to-day is a hospital service for that large class made up of those who, while unable to afford the usual expensive private service, and too proud to accept the charity and the publicity of the open ward, are willing and able to pay for a service somewhere between these extremes. How is this to be provided? It is manifestly not a proper tax upon the funds given for the poor, and it cannot be considered the duty of the city or of the State to provide such a service, the only reason for which is the pride of the individual, although it is a pride to be encouraged. This service must be so regulated as to be self-supporting, or else it must be provided for by special endowment. This is an important question, and we shall be called upon for the answer.

Another problem which I hesitate to mention, but which is so important that it cannot be passed by, is the matter of the training of nurses. We must all recognise that the trained nurse is to-day called upon to play an ever-increasing part in our civic and social life, and that as a factor in all public-health work her importance can hardly be over-estimated. My only plea is for more general recognition on the part of hospitals that the training school for nurses can no longer be regarded as simply a means to an end in the business of the hospital, that the hospital has a distinct duty to perform in the education of nurses, that it is a part of the educational function of the hospital and represents a great public service.

### Doctor and Nurse.

In a recent address to the nurses of the Johns Hopkins Hospital, Dr. William H. Welch expressed himself in words of great import, saying in part:

> The training school of the hospital supplies the needs of the hospital in the most efficient and economical way possible, but the query may be raised as to whether the complete meeting of that demand of the hospital is in every way commensurate with the meeting of the full needs of the training schools. I do not for a moment suggest that the training school shall not meet the needs of the hospital. That, of course, is the best part of the training of the nurse; but perhaps the hospitals should look upon it as not merely an arrangement by which the nursing needs of the hospital can be supplied, but as an opportunity to train women for a very important profession.
>
> I think the time has come to make a very urgent plea for the adequate endowment on the educational side of the training school for nurses, for adequate physical equipment and an adequate educational staff, and that the nurse shall have the requisite time not only for studying and attending lectures, but for reading, because practical training in nursing, as practical training in everything else, is, after all, one-sided, and it rests upon certain fundamental principles, and these principles must be taught just the same as in the training for any other technical profession. One is deeply handicapped without the knowledge of the fundamental principles which underlie the practice of any profession.
>
> I have already emphasised my feeling for trained nursing as a profession, and as a profession distinct from that of the practitioner of medicine—intertwined with it, of course; but I am sure it is important for the doctor to realise that there are things which the trained nurse knows better, and he should recognise that the nurse is an expert in a profession which, while intertwined with that of medicine, is distinct from it. It is not to be thought of as simply subservient to the profession of the practice of medicine. It stands by its side.

As an Association we have had this subject under consideration in one form or another for several years. Your special committee reports this year. It has gone deeply into the subject and will present to you a carefully prepared report which merits your earnest consideration.

The out-patient department, or dispensary, has received much attention in recent years, and it deserves and is bound to receive more in the future. It is a much more important phase of hospital work than has been generally understood. We have looked upon it, too often, as merely a side issue, where a place could be found for those for whom nothing else was available. We have heard much about dispensary abuse, meaning the abuse of the privilege by unworthy patients; but we have heard too little about the abuse of the patients by the dispensary, meaning the waste of the patient's time, and careless and superficial advice and treatment. This department is of sufficient importance to demand the highest grade of service, else it should be discontinued, for our hospitals will, in the future, be judged quite as much by the grade of service rendered in the out-patient department as by that in the wards.

Here again there is urgent need for a service for that great class of self-respecting, self-supporting individuals who are ready and willing to pay for expert service if it is placed within their means. In this day of specialisation, when to get a competent opinion means to pass through the hands of three or four specialists, they are unable to meet the expense. To satisfy this need, there should be pay clinics for the people of moderate means, entirely distinct and separate from the so-called free dispensary, where a competent opinion may be had from as many specialists as may be necessary, at a moderate, inclusive fee. The Massachusetts General Hospital, so often a pioneer, has again led the way in this respect, and the experiment should be carefully watched by us all.

### Hospitals and Public Morals.

A hospital exists to serve the public, and, if our hospitals are to play their full part, more attention should be paid to the establishment of clinics for venereal diseases. This applies both to the in- and out-patient services. I need not do more than call attention to the fact that the hospital has a great opportunity in such clinics for broader service, not only to those who suffer from these diseases, but to the public at large, in preventing the spread of venereal disease, both by therapeutics and education.

What should be the attitude of the hospital toward the illegitimate child and its mother? Dr. George Walker, chairman of the so-called Vice Commission in Maryland, called attention to the fact that not only were physicians willing to sanction and often to arrange for the prompt separation of the illegitimate child from its mother immediately after birth and to place it in a foundling asylum, but that hospitals quite frequently were willing parties to such an arrangement, some even providing a woman who had the specific duty of making such arrangements. I think we may assume that Maryland is not alone in this. Dr. Walker also points out that the mortality in foundling asylums is generally high; in many as high as 90 per cent. In consideration of this fact and of the moral principle involved, I believe that no hospital should countenance the separation of the child and its mother for such purposes. We all know that many times, when the mother has no desire to keep the child at first, if made to do so for a short time she would not part with it for any consideration.

We have accustomed ourselves, or are doing so, to the Workmen's Compensation Acts, as they affect our hospitals. Health insurance and the effect of the same on hospitals is another problem which we shall be forced to consider. Health insurance is doubtless in the minds of most of us a thing to be desired. In any event, it will surely be with us before many years. The American Association for Labour Legislation is actively engaged in promoting this movement, and I believe we should appoint a committee at this session to study the subject, to the end that as a body we may be better informed on the question, and, if it should be our judgment, to co-operate in all efforts to arrive at sound conclusions and to establish sound principles as the basis of such insurance.

In conclusion, I desire to thank the Association for the honour which it has paid me by selecting me as its President. It is not an honour which one seeks, but, as an expression of your confidence and trust, it is deeply appreciated. I have tried to prepare for you an interesting programme, and I sincerely hope that this, the eighteenth conference of the American Hospital Association, may be truly helpful and entirely harmonious.

I wish also to thank the other officers of the Association and the various committees for their loyal and whole-hearted support and consideration. If this meeting shall prove a successful and profitable one, as I am sure it will, the credit will be due to them, and in large measure to the Local Committee. I thank you for your patience and your consideration.

